# Lifestyle factors moderate emotion–gait coupling in young adults

**DOI:** 10.1007/s00221-026-07321-x

**Published:** 2026-06-08

**Authors:** Danielle D. Wadsworth, Bahman Adlou, John L. Grace, Jerad Kosek, Christopher Wilburn, Wendi Weimar

**Affiliations:** 1 School of Kinesiology, Auburn University, Auburn, AL USA; 2 School of Health Sciences, University of Evansville, Evansville, IN USA

**Keywords:** Mood, Physical activity, Sleep, Nutritional status, Principal component analysis

## Abstract

The current study investigated whether physical activity, sleep quality, nutritional patterns, and their composite moderate mood-gait relationships in healthy young adults. Participants completed a baseline assessment and over the course of the semester up to five repeated walking gait and mood assessments. At baseline, participants completed self-reported measures of current physical activity levels (IPAQ), sleep quality over the past month (PSQI), and nutritional intake (24-hour dietary recall). Of the 16 participants enrolled, 15 completed baseline surveys, and all completed at least two gait assessments. Most participants demonstrated moderate-to-high physical activity levels, with 86.7% meeting aerobic and 73.43% meeting resistance training guidelines. Sleep quality varied widely. Moderation analyses, accounting for visit number, identified three significant interactions after false discovery rate correction, all involving the gait rhythm domain. Higher baseline physical activity levels (both total MET-minutes and WHO physical activity category) attenuated the relationship between positive emotions and gait rhythm, suggesting that active individuals maintain more stable gait timing regardless of emotional state. Higher protein intake strengthened the association between negative emotions and gait rhythm. Three additional interactions, including a lifestyle composite score, showed trend-level effects (raw *p* < .012) warranting replication. These findings highlight the influence of lifestyle factors on emotion-related gait variability and indicate how modifiable health behaviors may influence emotion-locomotion coupling.

## Introduction

The relationship between emotional states and human locomotion represents a fundamental aspect of embodied cognition, with growing evidence demonstrating domain-specific influences of mood on gait parameters (Winkielman et al. 2015). Naturally occurring mood states influence gait in domain-specific ways, with negative emotions associated with alterations in temporal organization and increased spatial variability, while positive emotions are linked to more stable, rhythmic movement patterns (Adlou et al. 2025). These findings highlight that emotion-locomotion coupling extends beyond global changes in speed, reflecting more nuanced alterations in coordination and movement organization, with implications for both theoretical models of embodied emotion and applied movement-based assessment (Winkielman et al. 2015; Elkjær et al., 2022).

Walking gait provides an ideal paradigm for investigating emotion-locomotion relationships due to its reliance on both automatic motor processes and sensitivity to cognitive and emotional influences (Fawver et al. 2015). As established by Troje (2002) and further developed by Lord et al. (2013), gait can be decomposed into distinct domains, including phase organization, variability, rhythmicity, temporal parameters, and spatial components, each of which may respond differently to emotional states (Troje 2002; Lord et al. 2013). Prior work demonstrates substantial inter-individual variability in emotion-gait coupling, particularly within the phase organization domain, which exhibited markedly greater between-participant variance compared to other domains (Adlou et al. 2025). Utilizing the approach-avoidance motivational framework, positive emotional states facilitate approach behaviors characterized by increased gait speed and step length, while negative states evoke withdrawal behaviors reflected in reduced pace and increased cautiousness (Homagain and Martens 2023; Lebert et al. 2024). However, different negative emotions (e.g., anger, shame, guilt) produce distinct alterations in gait parameters rather than uniform changes, highlighting the need for emotion-specific investigations beyond simple valence categorizations (Adlou et al. 2025; Boudarham et al. 2013).

Despite growing evidence for mood-gait relationships, critical knowledge gaps remain regarding factors that might moderate these associations. Notable Adlou (2025) and colleagues showed inter-individual variability in the association between mood states and gait parameters. In particular, the phase organization domain exhibited the largest between-participant random intercept variance (4.870; SD = 2.21) among all five gait domains identified through PCA, substantially exceeding the variances observed for the remaining domains (range: 0.11–0.98; Adlou et al. 2025). Because domain scores are derived from standardized component scores, these values are on a unitless scale, and the magnitude of the phase domain variance suggests the presence of important but unexplored moderating variables. Three modifiable lifestyle domains: physical activity, sleep, and nutrition represent particularly promising candidates for moderating emotion-locomotion coupling, as each influence both mood regulation and motor control through parallel neurobiological pathways (Faulkner and Biddle 2013; Loprinzi et al. 2013; Michalak et al. 2009).

The SPAN framework (Sleep, Physical Activity, Nutrition) recognizes these lifestyle behaviors as interrelated and co-occurring, often clustering to produce synergistic effects on both physical and psychological functioning (Stamatakis et al. 2025). Each of these behaviors has independently been associated with both emotional regulation and motor performance. Regular physical activity is linked to improved gait stability and more efficient movement patterns, alongside enhanced emotional well-being (Schuch et al. 2016; Hollman et al. 2011). Individuals who engage in habitual physical activity tend to exhibit more stable and efficient movement patterns alongside enhanced emotional regulation and resilience, suggesting a potential buffering effect of physical activity on mood-related alterations in gait (Hollman et al. 2011; Stubbs et al. 2018; Hyde et al. 2011), This may be particularly evident in the relationship between positive affect and gait rhythm. Similarly, sleep quality is closely linked to both emotional functioning and motor performance, with poor sleep associated with impairments in emotional regulation, increased affective reactivity, and decrements in coordination and gait stability (Vanderlind et al. 2014; Van der Werf et al. 2009). Nutritional patterns, including overall diet quality and macronutrient balance, have also been linked to both mood states and functional performance outcomes (Hibbeln et al. 2018; Gibson & Bass, 1999; Sinclair et al. 2015; Hibbeln et al. 2018). Nutritional intake may also contribute to motor stability, as adequate protein and overall macronutrient balance support neuromuscular function and physical performance, (Phillips et al. 2016), with higher protein consumption related to walking speed (Coelho-Junior et al. 2019). Together, these findings suggest that lifestyle behaviors may function as moderators of emotion-locomotion coupling, either buffering or amplifying mood-related changes in gait.

Despite growing evidence for mood-gait relationships, limited research has investigated whether modifiable lifestyle behaviors influence these associations. This gap is particularly relevant in young adult populations, where physical activity, sleep, and dietary behaviors are highly variable and tend to co-vary (Laska et al. 2009), aligning with the SPAN framework (Stamatakis et al. 2025). Understanding how these lifestyle factors interact with emotional states to influence motor behavior may provide a more comprehensive account of embodied emotion.

Using principal component analysis to identify distinct gait domains and linear mixed-effects models to account for individual differences, several theory-driven hypotheses were tested:

1. Higher baseline physical activity would attenuate the relationship between positive mood states and gait rhythm parameters.
2. Poorer sleep quality would amplify the relationship between negative emotions and gait variability.
3. Higher protein intake would buffer the effect of mood on spatial gait asymmetry.
4. A combined lifestyle quality score would demonstrate stronger moderation of mood-gait relationships than individual lifestyle components.

By examining these moderation effects, this study’s findings advance an understanding of the complex interplay between lifestyle behaviors, emotional states, and motor control, potentially informing both theoretical models of embodied cognition and practical applications in health behavior interventions and assessment considerations.

## Methods

### Participants

To be able to participate, individuals had to be enrolled in one of two sections of a required kinesiology course at a large public university and have the physical ability to walk across an instrumented walkway. Recruitment took place at the start of the academic term and was conducted by a research team member unaffiliated with course instruction. Participants who signed the informed consent form and met study eligibility were enrolled in the study (initial sample *n* = 16). Those who completed the study were compensated with five bonus points on their final course test. All procedures described herein were approved by the Institutional Review Board and conformed to the standards set by the latest revision of the 1964 Declaration of Helsinki.

### Procedures

A repeated-measures design was executed to examine whether baseline lifestyle behaviors moderate the relationship between momentary mood states and gait parameters. The study protocol included: (1) comprehensive baseline assessment of lifestyle behaviors at the beginning of the semester, and (2) repeated assessments of gait and mood. At the outset of the study, participants received an email containing a baseline survey to assess demographic and anthropometric data, including age, sex assigned at birth, gender identity, race, height, weight, mood, current physical activity participation, self-reported sleeping behavior, and self-reported food consumption. After completing the baseline assessment, participants were randomly scheduled for five data collection sessions distributed across the semester, during which only gait and mood were evaluated. Each Monday evening, selected individuals were notified via email of their assigned session for that week. On Tuesday, they completed the gait assessment by walking along a sensor-equipped walkway located in a quiet area near the classroom. Immediately afterward, they completed a mood questionnaire using an electronic device supplied by the research team. This sequence was repeated weekly, and each participant was randomly scheduled to complete five total gait and mood assessments over the course of the semester.

### Measures

#### Gait

Spatiotemporal gait parameters were collected using the OptoGait photoelectric system (Microgate, Bolzano, Italy). The system consisted of two parallel 4.9-meter bars positioned 1.22 m apart, each containing 96 LEDs (1.041 cm resolution) that transmit (TX bars) and receive (RX bars) infrared signals. Interruption of these signals during walking enables precise detection of foot placement timing and position.

Participants performed three walking trials at their self-selected “natural” walking speed, simulating their typical campus walking pace. To ensure measurement of steady-state gait, participants began walking facing the same direction, three steps before entering and continued three steps after exiting the instrumented pathway. The first and final steps within the measurement zone were excluded from analysis to eliminate acceleration/deceleration effects (Kuo and Donelan 2010). Data were collected at 1000 Hz using OptoGait software (Version 1.13.17.0). During all walking trials, only two members of the research staff was present and the walkway was free of distractions.

Gait speed was calculated using the spatiotemporal parameters obtained from the OptoGait system. The calculation utilized step length and step time data to compute gait speed in meters per second (m/s) using the following:

$$\:Gait\:Speed=\frac{\left(Step\:Length*\:Cadence\right)}{60}$$

where step length is in meters and cadence (steps per minute) is derived from step time. Step length was converted from centimeters to meters, and cadence was calculated as 60 divided by step time in seconds. This method ensures consistent gait speed calculation across all trials and participants.

To account for anthropometric differences, spatial parameters were normalized to participant height (Hof 1996), and gait speed was normalized using the dimensionless Froude number (Fr = v²/gL, where v = gait speed, g = gravitational constant, L = leg length).

Twenty spatiotemporal parameters were collected during walking trials, including temporal parameters (e.g., step time, stance time, swing time), spatial parameters (e.g., step length, stride length), gait speed, phase parameters (e.g., single support, double support percentages), and variability measures (coefficient of variation for each parameter). These variables were selected based on established frameworks for gait analysis in adults (Lord et al. 2013) and validated protocols for comprehensive gait assessment (Hollman et al. 2011).

### Mood

Mood was evaluated by measuring participants’ current levels of both positive and negative emotional states. Using a 4-point Likert scale ranging from “not at all” (1) to “completely” (4), participants rated how much they felt emotions such as sadness, disgust, anger, guilt, shame, frustration, happiness, excitement, and alertness at the time of assessment (Mata et al. 2012). Completion of this survey typically took about two minutes. Positive mood (POS) was represented by the combined ratings of happiness, excitement, and alertness, while negative mood (NEG) was derived from ratings of frustration, sadness, disgust, anger, guilt, and shame. Composite scores were calculated by summing the relevant item scores and then rescaling the totals to align with the 1–4 range used in analysis. Prior research has demonstrated strong internal consistency for these scales, with Cronbach’s alphas of 0.92 for negative affect and 0.83 for positive affect (Mata et al. 2012). In the present study, reliability for the negative mood composite remained high (α = 0.88); however, internal consistency for the positive mood scale was comparatively low (α = 0.52), potentially due to fluctuating situational factors (e.g., varying academic activities or time-of-day differences) that influenced participants’ positive emotional responses. This pattern is consistent with prior findings that negative mood states tend to exhibit greater internal consistency than positive mood states (Han and Wang 2022), potentially because the three positive affect items (happiness, excitement, alertness) represent distinct facets of positive experience that may fluctuate independently in a college student sample.

### Baseline lifestyle behavior physical activity

Physical activity was assessed using the validated leisure-time section of the International Physical Activity Questionnaire-long form (IPAQ-long; Craig et al. 2003) at baseline. The IPAQ-long has demonstrated acceptable reliability and modest validity in adults aged 18–65 years. Specifically, test–retest reliability has been reported as approximately *r* = .80, indicating consistent responses over time, while criterion validity relative to objective measures (e.g., accelerometry) is lower, typically around *r* = .30 (Craig et al. 2003). These values are derived from multinational adult samples and are not specific to a particular age subgroup within this range. Participants reported frequency (days/week) and duration (minutes/session) of four activity categories during the previous seven days, prior to the baseline measure data time point: leisure walking, moderate-intensity activities, and vigorous-intensity activities. Additional questions that mirrored questions for moderate and vigorous activity were added to assess resistance training (Jones et al. 2025). Following established IPAQ scoring protocols (Craig et al. 2003), the following were calculated:

1. Activity-specific MET-minutes/week: Frequency × duration × intensity, where intensity values were: walking (3.3 METs), moderate activities (4.0 METs), vigorous activities (8.0 METs), and resistance training (5.5 METs).
2. Total MET-minutes/week: Sum of all activity-specific MET-minutes.
3. Guideline adherence indicators:

- Aerobic guideline adherence (binary; Bull et al. 2020; Mehl et al. 2023): Participants were classified as meeting aerobic guidelines if they reported (a) ≥ 150 min/week of moderate-intensity activity, (b) ≥ 75 min/week of vigorous-intensity activity, or (c) equivalent combination, where vigorous activity minutes were counted as double (i.e., moderate-equivalent minutes = moderate minutes + 2 × vigorous minutes; guideline met if total ≥ 150 min/week).
- Resistance guideline adherence (binary): ≥2 days/week of resistance training.

4. WHO physical activity category (ordinal; Bull et al. 2020): (1) Insufficient (< 150 moderate-equivalent min/week), (2) Sufficient (150–299 min/week), or (3) Highly Active (≥ 300 min/week).

### Baseline sleep quality

Sleep quality was assessed using the Pittsburgh Sleep Quality Index (PSQI; Buysse et al. 1989), a 19-item validated instrument measuring sleep quality and disturbances over the previous month. Participants manually entered responses to the initial five items, which asked for typical bedtime, time to fall asleep, wake-up time, total hours slept, and time spent in bed. The subsequent 14 items evaluated the frequency of various sleep-related difficulties, such as reliance on sleep medication and experiences of daytime drowsiness, using a 4-point scale ranging from 0 (not during the past month) to 3 (three or more times per week), with higher values indicating more frequent issues. The PSQI yields seven component scores (subjective sleep quality, sleep latency, sleep duration, habitual sleep efficiency, sleep disturbances, use of sleep medication, and daytime dysfunction) that sum to a global score (range: 0–21, higher scores indicating poorer sleep quality).

For moderation analyses, the following were utilized:

1. Global PSQI score (overall sleep quality) - a composite score ranging from 0 to 21 reflecting a person’s overall sleep quality over the past month.
2. Sleep efficiency percentage (time asleep/time in bed) - percentage of time spent in bed that a person actually spends sleeping.
3. Sleep disturbances component score - how frequently a person experiences various sleep-disrupting events during the past month rated on a 4-point scale from 0 (no sleep disturbances in the past month) to 3 (three or more sleep disturbances within the past month).

### Baseline nutrition

Nutritional intake was assessed using a validated 24-hour dietary recall protocol. Participants reported all food and beverages consumed during the previous day, including portion sizes and preparation methods. Nutritional composition was analyzed using USDA’s Food and Nutrient Database for Dietary Studies open-source software (https://reedir.arsnet.usda.gov). For moderation analyses, the following were extracted:

1. Total protein intake (g/day).
2. Total carbohydrate intake (g/day).
3. Total fat intake (g/day).
4. Protein-to-carbohydrate ratio.

### Composite lifestyle score

Following established frameworks for integrated lifestyle assessment (Garcidueñas-Fimbres et al. 2024; Hu et al. 2024), a comprehensive lifestyle quality composite was developed by combining standardized scores from three key behavioral domains:

$$ \begin{gathered} \left( { - {\mathrm{1}} \times {\text{ standardized sleep quality }} + {\text{ standardized total MET}}} \right. \hfill \\ \left. {\quad \quad - {\text{minutes }} + {\text{ standardized protein intake}}} \right)/{\text{ 3}} \hfill \\ \end{gathered} $$

Sleep quality was reverse scored so that higher values on all components indicate healthier behaviors, consistent with established practice in composite lifestyle indices. This standardization approach ensures equal weighting across domains while maintaining consistent directionality, where higher composite scores represent better overall lifestyle quality. This approach aligns with recent multicomponent lifestyle models that emphasize analyzing health behaviors as an integrated system rather than in isolation. This study’s methodology draws on the SPAN (Sleep, Physical Activity, and Nutrition) framework (Stamatakis et al. 2025).

### Statistical analysis

#### Gait dimensionality reduction

To reduce the dimensionality of the numerous gait parameters while preserving their underlying structure, a principal component analysis (PCA) with varimax rotation was deployed following established protocols in gait research (Adlou et al. 2025; Lord et al. 2013). The Kaiser-Meyer-Olkin measure confirmed sampling adequacy (KMO = 0.799). Five principal components with eigenvalues > 1.0 were retained, collectively explaining 84.67% of variance (Table 1). Component scores were calculated for each participant and standardized (z-scored) for subsequent analyses.

**Table 1:** Gait Domain Classifications Based on Principal Component Analysis

Domain	Variance Explained	Key Parameters
Temporal Phases (RC1)	39.57%	Stance%, swing%, single/double support%
Support Variability (RC2)	16.80%	Single/double support CV
Temporal Variability (RC3)	11.89%	Stance/swing time CV
Rhythm (RC4)	8.96%	Step/stance/swing time
Spatial Asymmetry (RC5)	7.45%	Step/stride length CV

### Moderation analysis

Based on previously established mood-gait relationships (Adlou et al. 2025), a focused moderation testing approach was implemented targeting theoretically relevant mood-gait pairs. For each pair, whether baseline lifestyle factors moderated the relationship was tested using linear mixed-effects models with participant as a random effect.

The moderation effect was tested by comparing a reduced model:

$$ \begin{aligned} {\mathrm{gait}}\_{\text{domain }}\sim & {\text{ mood }} + {\text{ moderator }} + {\text{ visit}}\_{\text{num }} \\ + & {\text{ C}}\left( {{\mathrm{session}}} \right){\text{ }} + {\text{ }}\left( {{\mathrm{1}}|{\mathrm{participant}}} \right) \\ \end{aligned} $$

to a full model that included the interaction term:

$$ \begin{aligned} {\mathrm{gait}}\_{\text{domain }}\sim & {\text{ mood }} + {\text{ moderator }} + {\text{ mood }} \times {\text{ moderator }} \\ + & {\text{ visit}}\_{\text{num }} + {\text{ C}}\left( {{\mathrm{session}}} \right){\text{ }} + {\text{ }}\left( {{\mathrm{1}}|{\mathrm{participant}}} \right) \\ \end{aligned} $$

where visit_num accounts for potential temporal trends in gait parameters across the study period, domain represents one of the five gait domains identified through PCA, Mood represents either a composite affect score (NEG/POS) or discrete emotion, Moderator represents one of the potential moderating variables, and (1|participant) accounts for within-participant clustering of observations. To account for potential temporal trends in gait parameters, visit number was included as a covariate in all models. Each moderator was tested in a separate model to avoid multicollinearity among conceptually overlapping measures within the same lifestyle domain (e.g., total MET-minutes and WHO physical activity category).

To evaluate whether baseline moderator variables differed by sex, Mann-Whitney U tests were conducted comparing male and female participants on all continuous lifestyle measures. No significant sex differences were observed (all *p* > .22), supporting the appropriateness of pooling sexes in the moderation analyses.

Models were estimated using restricted maximum likelihood (REML). Significance of moderation was determined using likelihood ratio tests comparing models with and without the interaction term. The Benjamini-Hochberg procedure (False Discovery Rate, or FDR) was applied to control the false discovery rate across multiple comparisons. All continuous predictors were standardized prior to analysis to enhance interpretability of interaction effects.

A total of 88 moderation effects across all mood–gait–moderator combinations were tested, with visit number and session included as covariates in all models, focusing particularly on relationships between: (1) negative affect/emotions and rhythm/temporal phases, and (2) positive affect/happiness and rhythm/spatial asymmetry.

All analyses were conducted in Python (version 3.12) using statsmodels (0.13.2) and scikit-learn (1.0.2).

## Results

Of the 16 participants who consented to the study (10 females, 6 males), 15 completed the baseline survey assessment and all 16 completed gait assessments with varying compliance: 12 participants completed all five sessions, one completed four, and two completed two sessions. One participant was excluded from analysis due to insufficient matched mood–gait data, and one additional participant lacked baseline survey data, resulting in analysis samples ranging from 14 to 15 participants depending on the measure. The final moderation analysis dataset included 14 participants contributing 60 matched mood–gait observations. Participants (*n* = 15 who completed the baseline surveys) demonstrated moderate-to-high physical activity levels (mean total MET-min/week = 3463.67 ± 2613.21), with 86.7% meeting aerobic guidelines and 73.3% meeting resistance training guidelines. Baseline sleep quality was variable (mean PSQI Global Score = 6.27 ± 2.96), with scores ranging from 1 to 12. Detailed participant characteristics are presented in Table 2.

**Table 2:** Participant Characteristics, baseline Physical Activity Levels, Sleep Quality, and Nutritional Intake

	All (*n* = 15^*†*^*)*	Female	Male	*p* (sex)
*Demographics (n = 16)*				
Participants	16	10	6	
Height	67.35 ± 3.09 cm	66.26 ± 2.19 cm	71.58 ± 1.87 cm	
Weight	72.05 ± 11.14 kg	71.22 ± 10.49 kg	84.76 ± 14.81 kg	
*Physical Activity (n = 15; sex-disaggregated: n = 14* ^*†*^ *)*				
Total MET-min/week (*n* = 15)	3463.67 ± 2613.21	2829.1 ± 2614.3	4518.8 ± 2694.0	0.228
Meeting aerobic guidelines, %	86.7	75.0	100.0	
Meeting resistance guidelines, %	73.3	62.5	83.3	
Meeting complete guidelines, %	66.7	50.0	83.3	
*WHO PA Categories*				
Insufficient Activity, %	13.3	25.0	0.0	
sufficient Activity, %	20.0	37.5	0.0	
Exceeds Guidelines, %	66.7	37.5	100.0	
*Sleep Quality (n = 15)*				
PSQI Global Score (*n* = 15) Range: 1–12	6.27 ± 2.96	6.62 ± 1.77	6.00 ± 4.43	0.794
Sleep Efficiency, %	0.90 ± 0.10	0.84 ± 0.05	0.87 ± 0.10	0.361
Poor sleep quality (PSQI > 5), %	66.7	87.5	50.0	
*Nutrition (n = 14)*				
Protein intake, g/day (*n* = 14)	121.80 ± 77.52	113.43 ± 68.29	147.12 ± 86.13	0.366
Carbohydrate intake, g/day	203.89 ± 115.17	196.83 ± 109.33	229.47 ± 130.85	0.628
Fat intake, g/day	86.82 ± 35.12	77.09 ± 29.26	98.18 ± 40.57	0.366
Protein Carbohydrate ratio	0.70 ± 0.54	0.56 ± 0.11	0.93 ± 0.78	1.000

^†^Overall values reflect all baseline completers (*n* = 15 for PA and Sleep; *n* = 14 for Nutrition). Sex-disaggregated values reflect the 14 participants (8 F, 6 M) included in moderation analyses. Demographics data represents all enrolled participants (*N* = 16); physical activity and sleep quality data represent participants who completed baseline surveys (*n* = 15); nutritional data represent participants who completed food frequency questionnaires (*n* = 14). Physical activity was assessed using the International Physical Activity Questionnaire (IPAQ); sleep quality was measured using the Pittsburgh Sleep Quality Index (PSQI), with scores > 5 indicating poor sleep quality; nutritional intake was calculated from 24-hour dietary recall. p-values reflect Mann-Whitney U tests comparing female and male participants on continuous moderator variables. MET = metabolic equivalent of task; WHO PA = World Health Organization Physical Activity; PSQI = Pittsburgh Sleep Quality Index

### Moderation effects of lifestyle factors

Moderation analyses, with visit number and session included as covariates, revealed three significant interaction effects after FDR correction (*p* < .05), all involving the Rhythm gait domain (Table 3). Three additional interactions showed raw p-values below 0.012 but did not survive FDR correction and are noted as trends.

**Table 3:** Significant Moderation Effects of Baseline Lifestyle Factors on Mood-Gait Relationships

Mood	Moderator	Gait Domain	Interaction β	*p*-value	*p*(FDR)
NEG	Protein intake	Rhythm	0.124	0.0017	0.0493
Happy	Total MET-min	Rhythm	-0.124	0.0005	0.0209
Happy	WHO PA category	Rhythm	-0.211	0.0002	0.0180

Note: Models include visit number and session as covariates, with participant as a random intercept. p(FDR) values reflect Benjamini-Hochberg correction across all 88 moderation tests. Three additional interactions showed raw* p*-values < 0.012 but did not survive FDR correction: NEG × Lifestyle composite (β = 0.188, p(FDR) = 0.053), Ashamed × Lifestyle composite (β = 0.157, p(FDR) = 0.095), and Ashamed × Protein intake (β = 0.100, p(FDR) = 0.100)

### Physical activity and positive emotions

Physical activity negatively moderated the relationship between happiness and gait rhythm. Both WHO physical activity category (β = − 0.211, *p* = .0002, p(FDR) = 0.018) and total MET-minutes (β = − 0.124, *p* = .0005, p(FDR) = 0.021) attenuated the effect of happiness on gait rhythm. This suggests that physically active individuals maintain more stable gait timing regardless of positive emotional state.

### Protein intake and negative emotions

Higher baseline protein intake significantly amplified the relationship between negative affect and gait rhythm (β = 0.124, *p* = .0017, p(FDR) = 0.049). This indicates that participants with higher protein consumption demonstrated stronger coupling between negative emotional states and temporal gait parameters.

### Lifestyle composite and negative emotions

**Fig. 1:**
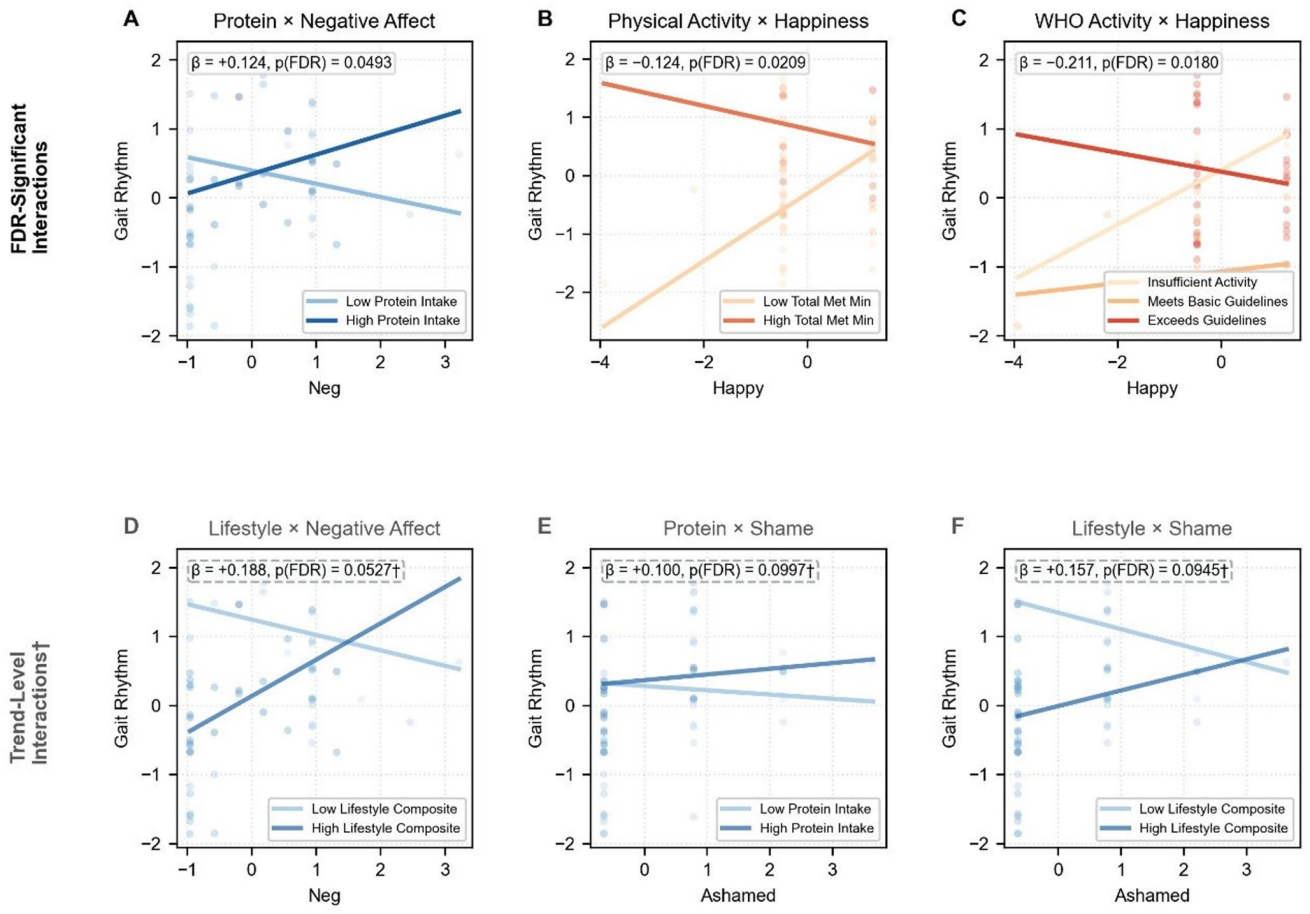
Moderation Effects of Lifestyle Factors on Mood–Gait Relationships. Top row (**A–C**): FDR-significant interactions (p(FDR) < 0.05). Bottom row (**D–F**): trend-level interactions (†raw *p* < .012, p(FDR) > 0.05). Lines show the relationship between mood (z-score normalized) and gait rhythm at high vs. low levels of the moderator variable (± 1 SD from the mean) for visualization purposes; statistical analyses used continuous moderator variables. Diverging lines indicate moderation effects, with interaction coefficients (β) and FDR-corrected* p*-values displayed for each panel. All models include visit number and session as covariates

The lifestyle composite score showed a nonsignificant moderation of the negative affect–rhythm relationship (β = 0.188, *p* = .004, p(FDR) = 0.053). Two shame-specific interactions effects were observed: Ashamed × Lifestyle composite (β = 0.157, *p* = .009, p(FDR) = 0.095) and Ashamed × Protein intake (β = 0.100, *p* = .011, p(FDR) = 0.100). These patterns suggest potential moderation by lifestyle factors for discrete negative emotions, though confirmation in larger samples is needed Fig. 1.

### Sensitivity analyses

Gait domain stability was assessed across visits. The Rhythm domain showed a statistically significant but substantively negligible temporal trend (β = − 0.002, *p* = .035); visit number was included as a covariate in all moderation models to account for this. Exclusion of participants with the fewest matched observations did not alter any findings (all three FDR-significant interactions remained significant with comparable effect sizes). Outlier diagnostics using Cook’s distance (threshold = 4/n) and studentized residuals revealed no extreme observations (maximum |studentized residual| = 2.69); all three significant interactions were robust to removal of influential observations.

## Discussion

This study investigated whether baseline lifestyle factors moderate the relationship between naturally occurring mood states and gait parameters in healthy young adults. After accounting for temporal trends by including visit number as a covariate and applying false discovery rate correction across all 88 moderation tests, the analysis identified three significant moderation effects, with three additional noteworthy interactions. These findings illuminate how modifiable health behaviors may influence emotion-gait coupling, with implications for both theoretical understanding and practical applications.

All significant and noteworthy moderation effects involved the Rhythm gait domain, with no effects observed for other domains (Temporal Phases, Support Variability, Temporal Variability, or Spatial Asymmetry). This domain specificity is noteworthy given that previous research shows mood effects were observed across multiple gait domains (Adlou et al. 2025), suggesting that while mood influences multiple aspects of gait, the moderation of these effects by lifestyle factors is selectively concentrated in the temporal organization of movement. Rhythm parameters (step/stance/swing time) depend heavily on cerebellar and basal ganglia function, which are regions influenced by both emotional states and lifestyle behaviors (Peterson and Horak 2016). Recent neuroimaging evidence indicates that the cerebellum serves as a key integration hub for emotional and motor processing (Ciapponi et al. 2023), and cerebellar functions extend beyond motor control to include emotional processing and regulation (Schmahmann et al. 2019), providing a neurobiological basis for the observed domain-specific moderation effects. The absence of moderation effects for spatial parameters, including the lack of support for the hypothesized moderation of spatial asymmetry, suggests that lifestyle factors may preferentially influence temporal rather than spatial aspects of emotion embodiment in locomotion. These same cerebellar and basal ganglia regions are part of the neural circuits that integrate emotional and motor processing (Takakusaki 2017), and further investigations using neuroimaging techniques could help elucidate why lifestyle factors selectively moderate emotion effects on temporal parameters but not spatial parameters.

### Physical activity and positive emotions

The significant negative moderation by physical activity measures on the happiness-rhythm relationship supports the first hypothesis. Both total MET-minutes (β = − 0.124, p(FDR) = 0.021) and WHO physical activity category (β = − 0.211, p(FDR) = 0.018) attenuated the effect of happiness on gait rhythm, indicating that physically active individuals exhibited weaker coupling between positive affect and temporal gait parameters, maintaining more consistent stride timing regardless of happiness level. The observed attenuation of happiness-gait coupling in physically active individuals may reflect exercise-induced adaptations in motor control networks (Schuch et al. 2016), potentially making gait timing less susceptible to emotional modulation. Previous research has demonstrated that exercise facilitates the release of neurotransmitters including serotonin, norepinephrine, and dopamine, which regulate both mood and motor function (Heijnen et al. 2016). Furthermore, habitual exercise produces region-specific adaptations in neurotransmitter systems that persist beyond individual exercise sessions, with cortical serotonergic changes lasting at least one week after discontinuing regular exercise (Heijnen et al. 2016). These sustained neurochemical adaptations may create a more stable neuromotor environment, reducing the relative impact of acute positive mood fluctuations on motor output.

The selective moderation of positive but not negative affect-gait coupling by physical activity may reflect distinct neurobiological pathways through which lifestyle factors influence emotion-gait coupling. Positive affect is associated with increased approach-oriented action tendencies and facilitation of motor responses, whereas negative affect is linked to avoidance behavior and greater interference with ongoing cognitive and motor processes (Phaf et al. 2014). Individuals who engage in regular physical activity may already exhibit more stable and efficient motor patterns, potentially reducing the incremental influence of positive mood on gait timing. In contrast, negative affect may influence gait through mechanisms such as attentional disruption and withdrawal-related behavior, which may be less susceptible to buffering by habitual motor automatization. This valence-specific pattern is consistent with evidence that positive and negative emotional states engage partially distinct motivational and neural systems in shaping action and movement (Phaf et al. 2014; Corr, 2013).

### Sleep quality and negative emotions

The second hypothesis, that poorer sleep quality would amplify the relationship between negative emotions and gait variability, was not supported. Despite robust literature linking sleep quality to both emotional processing and motor control (Palmer and Alfano 2017), no significant moderation effects were observed for any sleep quality metric in the current study. Although sleep quality showed moderate variability (PSQI: M = 6.27, SD = 2.96, CV = 48.0%), the PSQI captures habitual sleep patterns over the prior month, which may not reflect the acute sleep-related factors most relevant to emotion-motor coupling, particularly in this population. Future studies should incorporate both objective measures of sleep, such as actigraphy, and assessments of acute sleep patterns alongside habitual measures to comprehensively evaluate sleep’s potential moderating role in emotion-locomotion coupling.

### Protein intake and negative emotions

Higher protein intake strengthened rather than buffered the relationship between negative affect and gait rhythm (β = 0.124, p(FDR) = 0.049). This finding suggests that protein’s role in emotion-locomotion coupling may be more complex than anticipated. One possible explanation involves protein’s contribution to neurotransmitter synthesis: while protein provides amino acid precursors for mood-regulating neurotransmitters, individual differences in neurotransmitter metabolism could influence whether this heightens or dampens emotional responsiveness in motor circuits. Tryptophan (precursor to serotonin) and tyrosine (precursor to dopamine and norepinephrine) metabolism varies significantly between individuals, potentially explaining differential effects on emotion-gait coupling (Fernstrom et al. 2013; Fernstrom 2013). These individual differences manifest through several pathways, including genetic polymorphisms in enzymes such as tryptophan hydroxylase and tyrosine hydroxylase that affect neurotransmitter synthesis rates (Fernstrom 2013). Additionally, the ratio of tryptophan to large neutral amino acids influences serotonin synthesis, with higher protein intake potentially creating competition that paradoxically reduces serotonin availability in some individuals (Fernstrom et al. 2013; Heijnen et al. 2016). Furthermore, protein intake influences circulating levels of fibroblast growth factor 21 (FGF21), a hormone that coordinates feeding behavior and metabolism during periods of altered protein consumption (Laeger et al. 2014). These complex interactions between dietary protein, neurotransmitter synthesis, and neural circuits involved in both emotional processing and motor control may explain why higher protein intake strengthened rather than buffered the relationship between negative affect and gait rhythm.

Two additional shame-specific interactions showed trend-level effects that did not survive FDR correction: Ashamed × Protein intake (β = 0.100, *p* = .011, p(FDR) = 0.100) and Ashamed × Lifestyle composite (β = 0.157, *p* = .009, p(FDR) = 0.095). These patterns suggest potential moderation of discrete negative emotion-gait coupling by nutritional and composite lifestyle factors, though confirmation in larger samples is needed.

### Composite lifestyle score

The lifestyle composite interaction with negative affect showed a near-significant effect (β = 0.188, *p* = .004, p(FDR) = 0.053), with a numerically larger effect size than protein intake alone (β = 0.124). While this pattern is suggestive of synergistic effects among lifestyle domains, the composite did not survive FDR correction, and the fourth hypothesis is not supported in the current sample. Larger studies with greater statistical power are needed to determine whether integrated lifestyle quality demonstrates stronger moderation than individual components. This finding is nonetheless consistent with recent evidence that sleep, physical activity, and nutrition may exert synergistic rather than merely additive effects on neurobiological function, and aligns with the SPAN framework (Stamatakis et al. 2025), which emphasizes the importance of analyzing health behaviors as an integrated system.

### Embodied cognition framework

Considered together, the confirmed moderation effects reveal a valence-specific pattern. Physical activity attenuated the coupling between positive emotions and gait rhythm, while protein intake strengthened the coupling between negative emotions and gait rhythm. Within the embodied cognition framework, the negative moderation by physical activity suggests that habitual exercise creates a more stable neuromotor environment in which gait timing is more autonomous from emotional influence, a less “permeable” boundary between emotional and motor systems for positive states. Conversely, the positive moderation by protein intake suggests that certain nutritional factors may enhance the embodiment of negative emotional states, potentially through heightened neurotransmitter responsiveness in motor circuits. These bidirectional moderation patterns highlight the complexity of emotion-locomotion coupling and challenge simplistic models that assume uniform lifestyle-emotion-motor relationships.

These findings have several implications for research and practice. First, the differential moderation by physical activity versus protein intake underscores the importance of assessing multiple lifestyle domains when studying emotion-movement relationships. For health practitioners and exercise scientists, the results suggest that physical activity may promote emotional stability at the motor level, contributing to more consistent movement patterns regardless of emotional fluctuations, adding to the growing evidence base for exercise as a mood-regulatory intervention (Bernstein and McNally 2018).

For researchers designing clinical gait assessments, baseline lifestyle factors should be documented as potential confounds or moderators of emotion-gait relationships. For clinicians involved in rehabilitation, these findings suggest that lifestyle factors, particularly physical activity, may influence the extent to which emotional states manifest in gait patterns. In practice, this could help clinicians interpret variability in gait performance more contextually; for example, greater gait variability in less active individuals may partly reflect emotional fluctuations rather than solely underlying motor impairment. Conversely, more physically active patients may demonstrate greater consistency in gait timing across emotional states, which could be considered when evaluating progress or stability over time. As research in emotion- locomotion coupling evolves, such measures may eventually contribute to more holistic assessments that integrate emotional and behavioral context into motor evaluation, with potential applications for monitoring recovery or tailoring interventions. However, these findings remain preliminary and are based on a healthy young adult sample. Translation to clinical practice will require replication in clinical populations and the development of standardized, clinically feasible assessment protocols. Future intervention studies should examine whether lifestyle modifications can enhance emotional resilience through improved emotion-locomotion coupling, and whether multicomponent approaches targeting physical activity, nutrition, and sleep simultaneously produce stronger effects than single-behavior interventions.

### Limitations and future directions

This study has several limitations that should be considered when interpreting these findings. The small sample size (*N* = 16 enrolled; *n* = 14 in moderation analyses) limits statistical power and generalizability, potentially increasing the risk of Type II errors. Post-hoc power analysis indicated that the three FDR-significant interactions had large observed effect sizes (Cohen’s f² = 0.31–0.34) with statistical power exceeding 0.98; however, three additional interactions with raw p-values below 0.012 did not survive FDR correction after including visit as a covariate, and the marginal composite interaction (p(FDR) = 0.053) in particular warrants replication. The reduction from six to three FDR-significant interactions after including visit as a covariate reflects the conservative nature of multiple comparison correction with a small sample. Future studies should target 30 to 40 participants with five or more repeated measures to detect medium-sized moderation effects (f² = 0.10) with adequate power.

The homogeneous sample of college students restricted the range of lifestyle behaviors and mood states observed, limiting generalizability to broader populations. The internal consistency for the positive mood composite was below conventional thresholds (α = 0.52), potentially attenuating moderation effects involving positive affect, although this is consistent with previous literature (Han and Wang 2022). The sample size precluded sex-stratified moderation analyses; although no significant sex differences were observed in baseline moderator variables (all *p* > .22), future studies with larger, sex-balanced samples should examine potential sex differences in lifestyle moderation of emotion–gait coupling.

Baseline lifestyle assessments were conducted only once rather than repeatedly throughout the study period, limiting the ability to account for temporal changes in lifestyle behaviors. The observed moderation effects are consistent with baseline individual differences in lifestyle factors influencing emotion-locomotion coupling across the study period, but the temporal stability of these factors and their moderating influence cannot be confirmed from the current design. The reliance on self-report measures for all lifestyle assessments introduces potential recall bias, and college students may respond to self-report sleep measures differently than the general population due to normalized poor sleep patterns within this demographic (Palmer and Alfano 2017).

## Conclusion

This study provides novel evidence that baseline lifestyle factors differentially moderate the relationship between momentary mood states and gait parameters in healthy young adults. Physical activity attenuates the coupling between positive emotions and gait rhythm, while protein intake strengthens the association between negative emotions and gait rhythm. Trend-level evidence suggests that composite lifestyle quality may also moderate emotion-gait relationships, though this requires confirmation in larger samples. These findings highlight the importance of considering multiple health behaviors as an integrated system rather than isolated components when studying the complex interplay between lifestyle behaviors, emotional states, and gait. Future research should investigate whether lifestyle moderation effects differ between clinical and non-clinical populations, examine how acute versus chronic lifestyle behaviors differentially moderate mood-gait relationships and employ changes in lifestyle behavior over time.

## Data Availability

The datasets generated and/or analyzed during the current study are available from the corresponding author on reasonable request.
